# Electrochemical Sensors for Determination of Bromate in Water and Food Samples—Review

**DOI:** 10.3390/bios11060172

**Published:** 2021-05-27

**Authors:** Sheriff A. Balogun, Omolola E. Fayemi

**Affiliations:** 1 Department of Chemistry, Faculty of Natural and Agricultural Sciences, Mafikeng Campus, North-West University, Private Bag X2046, Mmabatho 2735, South Africa; sheriffawedabalogun@gmail.com; 2 Material Science Innovation and Modelling (MaSIM) Research Focus Area, Faculty of Natural and Agricultural Sciences, Mafikeng Campus, North-West University, Private Bag X2046, Mmabatho 2735, South Africa

**Keywords:** bromate, electrochemical sensors, electrochemical impedance spectroscopy, metal phthalocyanine, quantum dot

## Abstract

The application of potassium bromate in the baking industry is used in most parts of the world to avert the human health compromise that characterizes bromates carcinogenic effect. Herein, various methods of its analysis, especially the electrochemical methods of bromate detection, were extensively discussed. Amperometry (AP), cyclic voltammetry (CV), square wave voltammetry (SWV), electrochemiluminescence (ECL), differential pulse voltammetry and electrochemical impedance spectroscopy (EIS) are the techniques that have been deployed for bromate detection in the last two decades, with 50%, 23%, 7.7%, 7.7%, 7.7% and 3.9% application, respectively. Despite the unique electrocatalytic activity of metal phthalocyanine (MP) and carbon quantum dots (CQDs), only few sensors based on MP and CQDs are available compared to the conducting polymers, carbon nanotubes (CNTs), metal (oxide) and graphene-based sensors. This review emboldens the underutilization of CQDs and metal phthalocyanines as sensing materials and briefly discusses the future perspective on MP and CQDs application in bromate detection via EIS.

## 1. Introduction

Potassium bromate (KBrO_3_), a renowned oxidizing agent, has a huge reputation for being one of the best and least expensive dough improving substances in the baking industry. As such, its importance in the baking industry cannot be overemphasized. KBrO_3_ produced the desired result in baking by influencing the physical and chemical properties of macromolecules such as protein and starch often found in dough. Precisely, the viscosity, extent of gelatinization, swelling characteristics of the dough and disulfide linkage formation (in gluten proteins) are affected by the use of KBrO_3_ as an additive in bread baking [1]. Bromate has been found to be a product of water treatment due to bromide ion oxidation that occurs during ozonation.

Despite the importance of bromate in food production, numerous reports of its adverse effect on human health abound. Specifically, it has been reported to be connected to renal diseases, anemia, as well as peripheral neuropathy [1,2] if consumed beyond the allowed level of 25 µg L^−1^ by the world health organization (WHO, 1996). It has also been implicated in cancerous growths in laboratory animals. In addition, bromate in drinking water of mice and rats has been linked to an increase in cases of mesotheliomas of peritoneum, thyroid cell and renal tumor. Impaired auditory functions of humans and animals are also part of the scientifically confirmed result of a high level of bromate intake [3]. The United States Environmental Protection Agency (USEPA) and WHO have recommended 10 µg L^−1^ (0.078 µM) as the maximum acceptable level (MAL) as a result of its carcinogenicity [4]. Cancer cases as a result of bromate intake from water and food consumption have attained an alarming rate the world over, hence the need to control its concentration in bromate-containing water and food products to ensure consumer safety arises. It is noteworthy that the classification of bromate as a carcinogen in water and food was an outcome of toxicological examinations which confirmed bromate as a class B2 carcinogen (WHO, 1996) [5].

The determination of trace levels of BrO_3_^−^ requires the use of reliable, selective and very sensitive analytical techniques. High-performance liquid chromatography (HPLC), spectrophotometry, liquid chromatography, gas chromatography and ion chromatography [6,7,8,9] are the analytical techniques that have been used for bromate detection. However, multiple extraction, hydrolysis, special sample preparation, expensive and highly technical instrumentation, low sensitivity and high-temperature requirements for bromate extraction limit the application of these methods [10].

A model method for bromate determination would be expected to meet the following criteria:

1. Ability to determine BrO_3_^−^ down to a limit of detection that is 25% of the MAL
2. Short analysis time and cost
3. No sample pre-treatment
4. Method accessibility

The electrochemical method combines these features and is therefore considered one of the most suitable methods for bromate determination [11].

Electroanalytical methods utilize the relationship between an analyte’s concentration and potential (or current) change based on its chemical reactions to determine the quantity of an analyte. It is a quantitative means of analysis which basically depends on electrochemical processes in a medium or at the sensor–medium phase boundary. These electrochemical reactions are dependent on chemical composition, structural changes during analysis or concentration of the analyte.

Electroanalytical methods have some advantages over other analytical techniques. They allow the determination of various oxidation states of an element and not just the concentration of such element in solution. Low detection limits, characterization and information on the kinetics of a chemical reaction can be obtained through electroanalytical methods. Beyond these, this technique offers simplicity, rapid analyte detection and cost effectiveness. H_2_O_2_, hydrazine, dopamine, iodate, epinephrine, nitrite, glutathione, glucose, phthalates, oxalic acid, ascorbic acid, hydroquinone and citric acid are a few of the analytes that have been analyzed through electroanalytical methods [12,13].

A wide range of materials have been used for the development of chemically modified electrodes for bromate detection. Very low limit of detection has been achieved with these electrodes that ordinarily would not detect this analyte in the unmodified state [14,15]. This present review discusses the electrochemical methods and some other analytical techniques that have been deployed for bromate detection and future perspectives in the determination of the analyte. The performance in terms of sensitivity and detection limit of CNTs, graphene, polymers, quantum dots and some nanocomposite-modified electrodes for bromate detection are critically discussed in this review.

## 2. Electrocatalytic Reduction of Bromate

Electrocatalytic reduction of bromate produces different products, such as HBrO, Br_2_ and Br^−^. Table 1 below illustrates different reaction processes with their standard potentials (E° values vs. NHE). It could be deduced that the number of electrons involved and the standard potential values greatly determine the electrocatalytic reduction products. For complete electrocatalytic reduction of BrO_3_^−^ to Br^−^, the modified electrode must be able to produce 2 or 6 electrons, 4 electrons produce HBrO, while 5 electrons yield Br_2_. Common examples of electrocatalytic reduction of bromate with different electrochemical sensors are provided below.

Common examples of electrocatalytic reduction of bromate with different sensor materials are:

BrO_3_^−^ + 6H^+^ + 6e^−^ → ^POM^ Br^−^ + 3H_2_O (POM—Polyoxometalates)

Cd(II)–IL + e^−^ ↔ Cd(I)–IL

6Cd(II)–IL + BrO_3_^−^ + 6H^+^ ↔ 6Cd(II)–IL + Br^−^ + 3H_2_O

The number of electrons (n_o_) involved in the electrocatalytic reduction of BrO_3_^−^ can also be calculated from the scan rate study using Laviron’s equation [17]. For an irreversible electrode process, according to Laviron’s equation, the oxidation peak potential (Epa) is defined by the following equation:

(1) $$E{\mathrm{pa}}_{\;}=E{}^{\circ}+(\frac{RT}{\alpha n˳F})\;\ln\;\left(\frac{RTK{}^{\circ}}{\alpha n˳F}\right)+\left(\frac{RT}{\alpha n˳F}\right)\;\ln v$$

or

*E*_pa_ = K° − 2.3030(RT/αn_o_F) log(v) (2)

where α is the transfer coefficient, k^o^ is the electrochemical rate constant, n_o_ is the number of the electrons transferred, v is the scan rate and *E*^o^ is the formal potential. Other symbols have their usual meanings.

Another underlying factor that determines the BrO_3_^−^ reduction product is the pH of the solution. In a solid state, the electron transfer process is reversible and majorly pH-independent. The formation of Br^−^ is favored by a higher pH value because Br_2_ can exist in strong acid solution based on the reaction of

BrO_3_^−^ + 5Br^−^ + 6H^+^ → 3Br_2_ + 3H_2_O (3)

## 3. Bromate Electrochemical Techniques and Sensors

### 3.1. Determination of Bromate at Conducting Polymer-Based Modified Electrodes

Conducting polymers are electroactive polymeric materials which have recorded huge success as an important component of technological innovations such as anticorrosion coatings, batteries and electrochemical sensors. Electroanalysis of analytes in solution by conducting polymer-based sensors have proven to be very promising. This can be attributed to the interesting features of conducting polymers such as their high electrical conductivity and chemical stability [18].

An ECL sensor using poly (3-(1,1′-dimethyl-4-piperidinemethylene)thiophene-2,5-diyl chloride) (PTh-D) and nafion for the modification of Au electrode was fabricated by Li et al. [19]. Successful immobilization of PTh-D on the Au electrode in the presence of nafion was actualized due to the Au-S linkage between PTh-D and the Au electrode. The resultant ECL sensor had a linear relationship between BrO_3_^−^ concentration and ECL signal intensity (between 1 µM and 0.1 M) down to a detection limit of 1 µM. This sensor provided good recovery when applied for BrO_3_^−^ detection in drinking and river water.

Using a multiwalled CNT and 5,10,15,20-tetraphenyl-21H,23H-porphyrine iron (III) chloride (FeP) composite for glassy carbon electrode (GCE) modification, Salimi and his group [20] were able to present a sensing platform for BrO_3_^−^, chlorate and iodate detection. A pair of well-defined redox couples was obtained from cyclic voltammetry (CV) using this electrode. The fast electron transfer between FeP and MWCNT was confirmed by the rate constant (ks) and the surface coverage obtained for the electrode. The good electrocatalytic activity of this electrode towards BrO_3_^−^ reduction in acidic medium was characterized by the high stability, low limit of detection (LOD), good reproducibility, fast response time, wide linear dynamic range (LDR) and technical simplicity. The sensitivity, LOD and LDR obtained from BrO_3_^−^ detection by the electrode were 11 nA/µM, 0.6 µM and 2–150 µM respectively, via AP.

**Figure 1:**
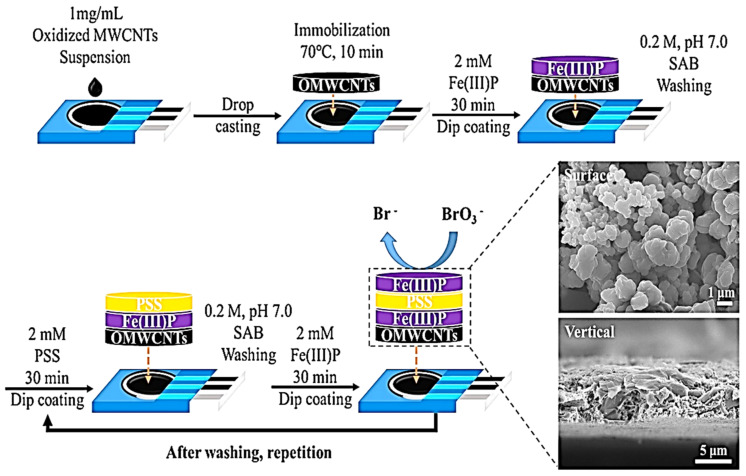
Schematic diagram of electrode fabrication of LBL process and FESEM images of (Fe(III)P-PSS)n-Fe(III)P-OMWCNTs/SPCE (adapted from [21] with permission).

Through a layer-by-layer (LBL) method of sensor fabrication, Yong-Gu and his team [21] assembled polyelectrolyte (polystyrene sulfonate, PSS), metalloporphyrin (FeP) and CNTs on screen-printed carbon electrode (SPCE) as shown in Figure 1. Using AP, LDR and LOD of 100 nM–2.5 µM and 43 nM respectively, were obtained. The authors were able to establish the fact that the LBL sensor is capable of rapid and selective BrO_3_^−^ detection in water samples. The electrode showed a good selectivity for BrO_3_^−^ in the presence of interferents (Mg^2+^, Ca^2+^, Na^+^, SO_4_^2−^, Cl^−^ and ClO_4_^−^), except HCO_3_^−^ that showed a noticeable interference effect. In comparison, this result has a better LOD than that in [20] using the same sensor material.

A simple method of BrO_3_^−^ determination in water and bread samples was presented by Wang et al. [22]. This was accomplished with the aid of a monolith column made of poly(glycidyl methacrylate-co-ethylene dimethacrylate) obtained via in-situ polymerization followed by quaternary amine modification. After a post-column reaction with KI at a wavelength of 352 nm, BrO_3_^−^ was detected. LOD and LDR of 1.5 and 5–30 µg/L respectively, were obtained within an analysis time as short as 8.5 with good standard deviation (n = 6, 0.043%).

The popular conducting polymer, poly(3,4-ethylenedioxythiophene) (PEDOT) prepared through the electropolymerization of its monomeric unit (PEDOT) and silicomolybdate (SiMo_12_O_40_^4−^), was applied for the modification of an electrode for BrO_3_^−^ detection by Balamurugan and Shen-Ming [3]. The resultant electrode had the capacity for fast propagation of charges in acidic medium and fast response time (<10 s). This fast response was ascribed to short penetration depth of BrO_3_^−^ through a very active polymeric film. The strong electrostatic interaction between PEDOT and polynuclear inorganic compound accorded the electrode its good chemical stability and reproducibility. The authors concluded that the electrode can accurately measure BrO_3_^−^ concentration over a LDR of 30–8000 µM and can also be applied for ascorbic acid (AA) detection.

Electrocatalytic reduction of BrO_3_^−^ in water sample was actualized by Ali et al. [23] using an electrode based on the electropolymerization of Ni-substituted polyoxometalate (POM) and pyrrole. The polymer films were made in various film thicknesses and characterized prior to the analyte detection. It is noteworthy that EIS confirmed the conductivity of this POM-doped polypyrole (Ppy) co-polymer film. The best of the polymer films in terms of stability and electrocatalytic activity towards BrO_3_^−^ reduction was used for BrO_3_^−^ detection in the water sample. The electrode offered a sensing platform with LOD and LDR of 0.2 µM and 0.1–2 mM, respectively.

Using an electrode modified with lanthanide-molybdate (LM) complex and Ppy film, Shaojun and his group of researchers [24] achieved a reproducible electrochemical detection of BrO_3_^−^. The investigation of the effect of pH on the electrochemical activity of the electrode towards BrO_3_^−^ detection revealed that the various forms of Ppy during the redox process influence the relationship between pH and the formal potential of LM-Ppy. The electrode was confirmed (CV studies) as a sensor with good potential for BrO_3_^−^ detection due to the relatively wide LDR (1–32 nM) obtained with the electrode. The results obtained here show a better LDR than that in [23].

An amperometric sensor targeted towards BrO_3_^−^ detection was developed by Li et al. [25]. This was actualized by the combination of Na_2_H_6_Co(H_2_O)O_39_.14H_2_O complex and poly(vinylpyridine) in the presence of a TiO_2_ sol. Similarly, an amperometric sensor made from a tungsten oxide film for BrO_3_^−^ electrochemical reduction was fabricated by Casella and Contursi [26]. Unfortunately, these sensors offered relatively high LOD for BrO_3_^−^ detection.

Another Ppy-based sensor for BrO_3_^−^ was prepared by Zou et al. [27]. The authors immobilized polyaniline (PANI) and Ppy on an electrode using POM as a dopant. The electrocatalytic properties of the POM were affected by the presence of the polymers. Some other POM-based sensors have also been reported in the literature [28,29].

GCE modified with MXene (lamellar Ti_3_C_2_T_x_) was fabricated by Rasheed et al. [30] for BrO_3_^−^ determination via differential pulse voltammetry (DPV). The good electrocatalytic properties of this sensor are reflected in the low LOD and wide LDR, of 41 nM and 50 nM–5 µM, respectively. The redox reaction between MXene and BrO_3_^−^ was confirmed by the formation of TiO_2_ at Ti_3_C_2_T_x_ surface during BrO_3_^−^ reduction. The electrode was also able to selectively detect BrO_3_^−^ in the presence of interferents (Br^−^, H_2_PO_4_^−^, HPO_4_^2−^, PO_4_^3−^, SO_4_^2−^, Cl^−^, NO_3_^−^ and ClO^−^). This electrode is one of the very efficient sensors for a water contaminant with a simple method of preparation. A better LOD was obtained with this technique compare with that in [19].

BrO_3_^−^ determination was also investigated with the application of another GCE modified with MWCNTs, PM and polydiallyldimethylammonium chloride (PDDA) through the LBL approach. Pang and his group [31] reported that PM was electropolymerized on PDDA/MWCNTs-modified GCE to obtain the working electrode. The excellent electrocatalytic activity of this sensor manifested in an extremely low LOD (20 nM) and response time (1.53 s), evaluated by the authors. The sensor also had a wide LDR (50–400 nM) and high sensitivity (13.81 mA cm^−2^ mM^−1^) towards BrO_3_^−^, which was achieved using the AP technique.

Sheen et al. [32] fabricated a sensor for BrO_3_^−^ detection by modifying gold electrode (GE) with 5, 10, 15, 20-tetrakis (4-methoxyphenylporphyrinato) (TMOPP) and Manganese (III) chloride (Mn(III)Cl). The resultant sensor TMOPPMn(III)Cl/GE was used for BrO_3_^−^ determination in the bread sample. This sensor showed good electrocatalytic activity towards BrO_3_^−^ at a pH of 7 in 0.1 M Na_2_SO_4_ solution, with LOD and LDR of 3.56 nM and 0.1–1 × 10^4^ µM respectively, using SWV techniques. The electrode also proved selective for BrO_3_^−^ in the presence of 100-fold excess of the interferents (glucose, sodium carbonate, sodium chloride, K^+^ and Ca^2+^).

### 3.2. Determination of Bromate with Carbon Nanotubes (CNTs)-Based Electrodes

CNTs have received wide attention in the field of nanotechnology due to their outstanding opto-electronic properties. Specifically, their biocompatibility, high reactivity, good conductivity and modifiable sidewall have made their application in sensors’ fabrication highly embraced in electroanalytical chemistry [33,34]. The success of such sensors has manifested in improved current response of biomolecules, inorganic compounds and some biological cells when CNTs are incorporated as part of a composite for the modification of an electrode. In addition, CNTs stand out as a sensing material as a result of their chemical stability, fast electron transfer kinetics and electrocatalytic activity towards a wide range of analytes in non-aqueous and aqueous media [34,35].

In agreement with the foregoing, Li et al. [36] presented an amperometric BrO_3_^−^ sensor based on MWCNTs and phosphomolybdic (PM) acid composite. This composite was applied for the modification of pyrolytic graphite electrode (PGE) for improved sensitivity of PGE towards BrO_3_^−^. Due to the synergy between the components of the composite, the sensor offered a fast response time (<2 s), wide LDR (5–15,000 µM) and a relatively low LOD (0.5 µM). An interference study showed that the common interferents (K^+^, Na^+^, NH_4_^+^, Ca^2+^, Mg^2+^, Zn^2+^, Cl^−^, Br^−^, I^−^, H_2_PO_4_^−^, HPO_4_^2−^, PO_4_^3−^ and SO_4_^2−^) do not interfere with the detection of BrO_3_^−^, except a few ions (CO_3_^2−^, NO_2_^−^, ClO_3_^−^, IO_3_^−^ and Fe^3+^) that exhibited interference with different degrees.

Similarly, a GCE modified with single-walled CNTs and Os (III) complex was fabricated by Salimi and his team [37] for BrO_3_^−^ detection. This electrode gave LDR and LOD of 1–2000 µM and 36 nM, respectively. The suitability of the electrode for BrO_3_^−^ detection was also characterized by good reproducibility, fast response time, technical simplicity and the reversibility of the redox couple.

**Figure 2:**
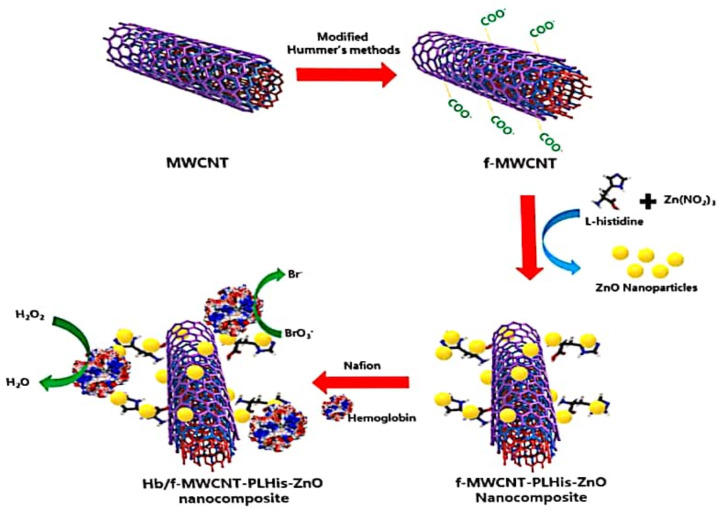
Schematic representation of the preparation of Hb/f-MWCNT–P-l-His–ZnO-modified electrode for the development of the bromate and H_2_O_2_ biosensor (adapted from [38] with permission).

A biosensor with dual function of BrO_3_^−^ and H_2_O_2_ detection was made available by Vilian and his group [38]. The biosensor was made (as illustrated in Figure 2) by immobilizing hemoglobin (Hb) on a composite made from the combination of functionalized MWCNTs, poly-L-histidine (P-his) and ZnO nanoparticles. The *ks* value obtained from this sensor was 5.16 s^−1^, while the surface coverage of Hb and response time were 1.88 × 10^−9^ mol cm^−2^ and <3 s, respectively. The LOD and LDR reported for this electrode via AP were 0.30 µM and 2–15,000 µM, respectively. Good stability and reproducibility are the advantages of this electrode. The sensor was applied for BrO_3_^−^ detection in urine, tap water and local river water, with good recovery.

Salimi and his group [39] presented another SWCNT, copper complex [Cu(bpy)_2_]Br_2_ and silicomolybdate immobilized onto glassy carbon (GC) electrode for electrochemical BrO_3_^−^ detection. The fabrication of the electrode SiMo_12_O_40_^4−^/[Cu(bpy)_2_]^2+^/CNT/GC was facilitated by the electrostatic interaction between the [Cu(bpy)_2_]Br_2_*/α*-SiMo_12_O_40_^4−^ and SWCNTs. The presence of SWCNTs brought about improved conductivity and porosity to the fabricated electrode. CV was used to study the electron transfer kinetics of the adsorbed redox couples, as well as the electrochemical behavior and the stability of the electrode. Consequently, this modified electrode was used for the amperometric BrO_3_^−^ detection. LOD, LDR and sensitivities of 1.1 nM, 0.01–20 μM and 6.7 nA nM^−1^ respectively, were obtained with this sensor.

Dan-dan et al. [40] achieved selective BrO_3_^−^ detection using a nanocomposite made from Pd nanoparticles and MWCNTs. CV showed reduction peaks for BrO_3_^−^ between potentials of 0.15 to −0.25 V. Using chronoamperometry (CA), a very wide LDR (0.1–40 mM), short response time (5 s) and high sensitivity (768.08 µA mM^−1^ cm^−2^) were reported for this electrode. This study confirmed that Pd/MWCNTs nanocomposite is a suitable sensing material for BrO_3_^−^ detection.

Another hemoglobin (Hb)-based electrode for BrO_3_^−^ determination was prepared by Li et al. [41] by immobilizing Hb on GCE modified with MWCNTs dispersed in PLL (MWCNTs-PLL). The modified electrode showed good electrocatalytic activity towards BrO_3_^−^ detection at a pH of 5.6. Using amperometry, LOD and LDR of 0.96 µM and 15–6000 µM respectively, were recorded for this electrode. The authors confirmed that the electrode can be used as a simple and accurate means of BrO_3_^−^ detection in real samples (mineral water). Vilian and his group [38] performed a similar study with a much lower LOD.

### 3.3. Determination of Bromate at Graphene/Graphene Oxide-Based Electrodes

Since the emergence of reports on the electrochemistry of graphene in 2008, graphene has attracted tremendous attention as a carbon nanomaterial for electrode fabrication as a result of its two-dimensional nature [42]. Successive years witnessed the abundance of publications on the modification of GCE with graphene produced via graphitic oxide chemical reduction [42,43,44,45,46]. These electrodes have been applied for detecting various analytes as electrochemical sensors in an oxygen reduction reaction as electro-catalyst [45].

Recently, graphene has been combined with a wide range of nanomaterials and polymers for the fabrication of sensors with high sensitivity towards a large number of analytes. This happened based on the fact that graphene has unique electrocatalytic, optical, physical and mechanical properties, such as high mechanical strength, large surface area, good electrical conductivity, high transparency and strong ambipolar electric field effect. The electrical conductivity of graphene, which enhanced the electron transport properties of graphene-modified sensors, stemmed from the sp2 hybridization and the presence of some oxygen-containing functionalities in graphene or an oxidized form of it. These attributes have contributed to the surge in popularity of graphene and its derivatives in electrochemistry and the nanotechnology world at large. This popularity manifested in the application of this material in capacitors, batteries, sensors high-frequency circuits, fuel cells and transparent conductive films [18,47].

Majid and his group [11] fabricated graphene oxide (GO)-modified GCE for BrO_3_^−^ determination. Therein, a GO and Pd nanocomposite was deposited on a clean GCE to obtain a working electrode tagged Pd-GO/GCE. Amperometry studies with this electrode gave a LOD of 0.10 µM over a LDR of 1–1000 µM. The author confirmed that no interference was observed with K^+^, Na^+^, NH_4_^+^, Mg^2+^, Zn^2+^, Cl^−^, H_2_PO_4_^−^, HPO_4_^2−^, NO_3_^−^, ClO_4_^−^ and PO_4_^3−^, except Br^−^ and Fe^3+^. Real sample analysis of BrO_3_^−^ detection was carried out with flour and bread samples with good recovery. The LOD reported here is higher compared with Sheen et al. [32].

GCE modification with *β*-cyclodextrin (*β*-CD) and graphene (Gr) for BrO_3_^−^ detection was described by Palanisamy et al. [48]. Hemoglobin (Hb) was further immobilized on the modified electrode (*β*-CD-Gr/GCE) to obtain a sensor with fast charge transport tendency. This electrode was characterized by high ks (3.18 ± 0.7 s^−1^), relatively wide LDR (0.1–176.6 µM) and low LOD (33 nM) at a potential of −0.33 V. The authors also reported that the electrode has good reproducibility and selectivity for bromate in the presence of interfering species (Mg^2+^, Fe^2+^, Fe^3+^, Ni^2+^, Ca^2+^ Cl^−^, Br^−^, l^−^, NO_2_^−^, NO_3_^−^ and lO_3_^−^).

**Figure 3:**
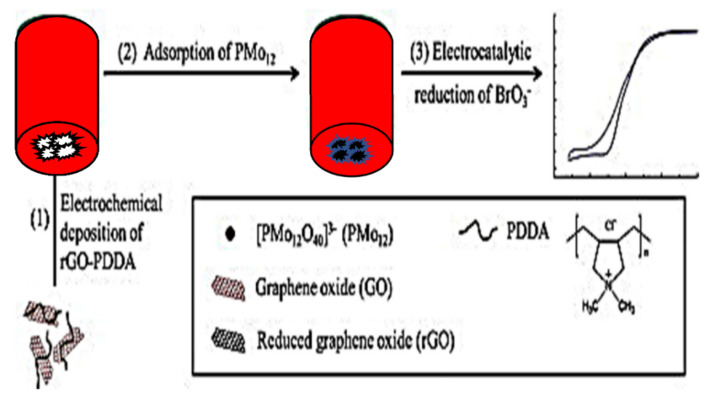
Schematic illustration of the principles of the fabrication of a PMo_12_@rGO-PDDA/GCE and the catalytic reduction of bromate (adapted from [16] with permission).

Ding et al. [16] presented a BrO_3_^−^ sensor made by the further modification of rGO-modified GCE with phosphomolybdate (PM) and poly(diallyldimethylammonium chloride) (PDDA). The process of fabricating rGO-PDDA/PMo_12_/GCE modified electrode involved three steps as illustrated in Figure 3. The stability of the electrode hinged on the electrostatic attraction between the cationic PDDA and the negatively charged PM. With CV, the electrocatalytic activity of the electrode towards BrO_3_^−^ reduction and its stability were established. This electrode had a wide LDR (0.02–10 µM) with high sensitivity (454 µA cm^−2^ mM^−1^). The same PDDA/PMo_12_ was also used by Pang and his group [31], but with a better result for LOD (20 nM).

Recently, Zhang et al. [49] accomplished a photocatalytic means of BrO_3_^−^ and ibuprofen (IBP) detection with GO and TiO_2_ doped with fluorine particles (FGT). At optimum condition, the photocatalytic degradation of BrO_3_^−^ and IBP fitted into Langmuir–Hinshelwood first-order kinetics. BrO_3_^−^ reduction to bromine was actualized by electron transfer, while the simultaneous consumption of BrO_3_^−^ and IBP inhibited electrons and hole recombination, thus making a huge utilization of the redox potentials of FGT. This study is proof that the FGT assemblage is an efficient means of quantifying selected water pollutants.

GO enables the BrO_3_^−^ formation when bromide-containing water undergoes ozonation, with yields up to double what could be obtained using only ozone. This was attributed to the increase in the amount of hydroxyl radical generated in the process. In order to reduce BrO_3_^−^ formation, Ye et al. [50] prepared an rGO (from hydrothermal treatment of GO)-supported CeO_2_. This nanocomposite was able to achieve a better inhibition rate (73%) than using only rGO. This study further revealed that the presence of Ce^3+^ on the composite is capable of quenching Br^−^ and BrO^−^ in order to inhibit BrO_3_^−^ formation.

### 3.4. Determination of Bromate at Metal/Metal Oxide-Based Modified Electrodes

The emergence of metal and metal oxide nanoparticles in electrochemical sensors’ fabrication was a consequence of the need to urgently fill the vacuum created by the individual use of polymers and carbon nanomaterials such as CNTs, Gr, GO and carbon quantum dots (CQDs). Metal and metal oxide nanoparticles have combined with these materials to address challenges such as the agglomeration of CQDs, stacking of Gr lamellae and adhesion of CNTs [51,52]. Consequently, metal and metal oxide nanoparticles have succeeded in functionalizing other materials. The nanocomposites formed in the process have been put to a wide range of practical uses because a composite combines the attributes of its components [53]. This combination often results in the formation of materials with improved biocompatibility, surface area, conductivity and electrocatalytic activity, which culminate in better electron transfer kinetics compared to that of individual materials [54]. It is noteworthy that nanomaterials have been used for sensor fabrication because they possess better chemical and electronic properties than bulk materials [55].

Ourari et al. [56] synthesized a Cu II-[N,N′-bis(2,5-dihydroxybenzylidene)-1,2-diaminoethane] (Cu II-DHB) electrode modified by carbon paste, which was used for the simultaneous detection of NO_2_^−^ and BrO_3_^−^ via amperometry and differential pulse voltammetry (DPV) techniques. Voltammetric studies revealed that the rate-determining step involved one electron, thus indicating that the process was purely diffusion-controlled. LOD and LDR of 1.5 and 2–14 nM respectively, were obtained for NO_2_^−^ via DPV, while LOD and LDR of 10 and 2–14 nM respectively, were obtained for BrO_3_^−^ via amperometry. The modified electrode exhibits a high selectivity for both nitrite and bromate in the presence of interferents (NO_3_^−^, Cl^−^ and SO_4_^−^), except lO_3_^−^ (due to its equal potential range with copper (II) Schiff base complex). This result showed that [CuII-DHB]-CPE is an effective electrochemical sensor for detecting bromate.

A new cadmium-ionic liquid-carbon paste electrode (Cd-IL/CPE) was fabricated for the simultaneous detection of trichloroacetic acid (TCA) and bromate by Zhuang and his team [57]. CV studies revealed that the fabricated electrode has good electrocatalytic activity towards TCA and BrO_3_^−^ reduction at pH 6.1 in 0.1 M B-R buffer solution. This electrode was used for electrochemical detection of BrO_3_^−^, with LOD, sensitivity and LDR of 3 nM, 496.15 μA μM^−1^ and 0.005–0.020 μM, respectively. The authors also reported that the electrode has a much lower detection limit than the earlier reports of References [20,35], in which other modified electrodes were used.

In a bid to present a platform for simultaneous bromate, iodate and chlorate detection, Arumugam et al. [58] fabricated a silver-phosphomolybdate-polybenzidine nanocomposite (Ag/PMo_12_/PBz) on a glassy carbon electrode (GCE). An amperometric study showed that the Ag/PMo_12_/PBz/GCE electrode has a better sensitivity and a much lower LOD towards BrO_3_^−^ than ClO_3_^−^ and IO_3_^−^. The best electrocatalytic activity of the electrode towards BrO_3_^−^ was achieved in 1 M H_2_SO_4_ solutions. LOD and LDR of 86.3 nM and 2.34 nA μM^−1^ respectively, were obtained with this electrode under optimal conditions. The ease of preparation, fast response as well as mechanical and electrochemical stability are the major advantages of this electrode. Fortuitously, this LOD is much lower than the one previously reported for another amperometric sensor prepared by Li et al. [36].

A nanocomposite made from the cross-linkage of chitosan (CHT) with a zero-valent cobalt 2,6-pyridine dicarboxylic acid (ZVCo-PDCA-CHT) was developed by Akinremi et al. [59] for the determination of BrO_3_^−^ in water. The working principle of the technique relied on the reduction of Co (II) with NaBH_4_ for a resultant BrO_3_^−^ reduction. The cross-linkage of CHT was facilitated by the 2,6-pyridine dicarboxylic acid (PDCA). With this composite, 99% BrO_3_^−^ reduction in water was accomplished within 1 h, while a 65% reaction completion was reported with PDCA cross-linked CHT.

**Figure 4:**
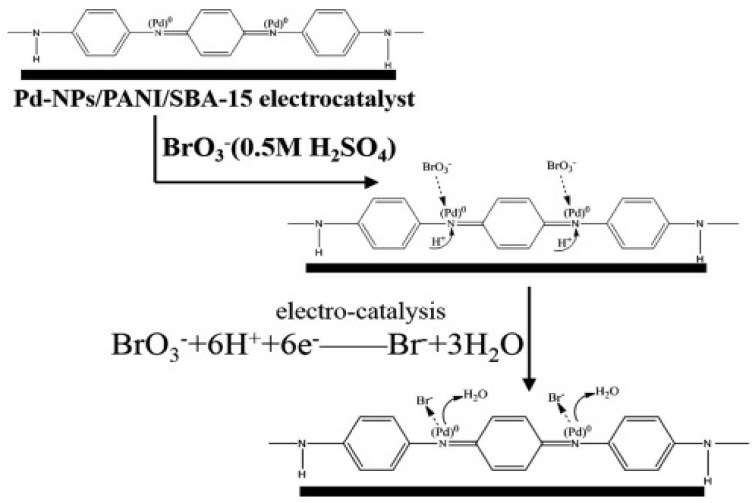
Schematic representation of the proposed electrocatalytic reduction process of BrO_3_ at the Pd-NPs/PANI/SBA-15 interface (adapted from [60] with permission).

**Figure 5:**
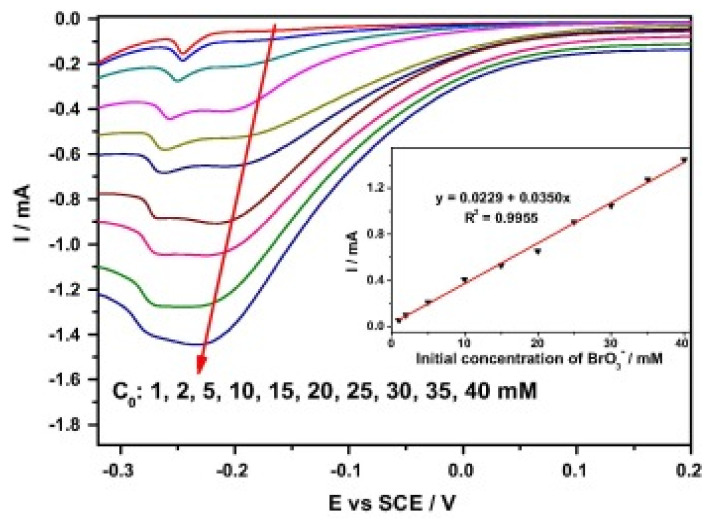
A series of cyclic voltammograms of Pd-NPs/PANI/SBA-15 in 0.5 mol L^−1^ H_2_SO_4_ with different BrO_3_ concentrations of 1, 2, 5, 10, 15, 20, 25, 30, 35 and 40 mmol L^−1^ at a scan rate of 20 mV s^1^. The inset in the picture shows the plotting of the corresponding reduction peak current vs. the BrO_3_ concentration (adapted from [60] with permission).

Through an in-situ approach, a sensor for BrO_3_^−^ detection was fabricated by Sun et al. [60] by the deposition of Pd nanoparticles (PdNPs)-coated PANI on a mesoporous SBA-15 support. A stepwise description of the electrocatalytic reduction process of BrO_3_^-^ at the Pd-NPs/PANI/SBA-15 interface is given in Figure 4 while Figure 5 illustrating the cyclic voltammograms of the modified electrode in 0.5 mol L^−1^ H_2_SO_4_ with different BrO_3_ concentrations. The electrode showed good electrocatalytic activity towards BrO_3_^−^ reduction over a potential window of 0.12 to −0.22 V. The amperometric studies revealed that the electrode has a LOD and a very wide LDR of 5 and 8–40,000 µM, respectively. The stability of the electrode was confirmed by 200 cycles of CV scans. The electrode showed great potential for practical BrO_3_^−^ detection in real samples. It is noteworthy that the sensitivity of the electrode was ascribed to the availability of large nitrogen sites on the composite for PdNPs anchorage, improved surface area of the electrode due to the presence of SBA-15 and the presence of abundant H^+^ for BrO_3_^−^ reduction. 

Sun et al. [60] enumerated three factors that led to the high sensitivity of Pd-NPs/PANI/SBA-15 for BrO_3_ reduction. These include:

1. The large number of PANI/SBA-15 nitrogen sites available for anchorage of Pd-NPs that ensures a large quantity of uniformly dispersed small Pd-NPs.
2. The successful incorporation of mesoporous SBA-15 significantly increases the effective electrode surface and electrolyte diffusion velocity.
3. The strong acidity of the medium that provides abundant H^+^ for the BrO_3_ electroreduction reaction.

Cheng et al. [61] fabricated a gold-rhodium AuRh nanoparticle-modified GCE for BrO_3_^−^ detection. The small particle size of the AuRh nanoparticles was partly responsible for the reduction in the over potential and the emergence of a well-defined peak for BrO_3_^−^ detection in the presence of PBS (pH 7.0) in a CV experiment. This sensor offered LOD and LDR of 1.0 mM and 1–26 µM, respectively.

The determination of the BrO_3_^−^ content of hair care products was carried out by Chen and his group [62] using a CuO nanoparticle (CuO NP)-modified SPCE. This was accomplished by the deposition of CuO NPs on SPCE, which enhanced the reduction of BrO_3_^−^ in weak acidic media. The CuO/SPCE was incorporated into a flow injection analysis system for the development of a very sensitive platform for BrO_3_^−^ detection. LOD and LDR of 3.5 µg L^−1^ and 0.01–300 mg L^−1^ were reported for this system. This technique also showed good selectivity for BrO_3_^−^ in the presence of interferents (F^−^, Br^−,^ Cl^−^, ClO_4_^−^, SO_4_^2−^ and NO_3_^−^, neither the sensitivity nor the response time of the electrode was affected by the addition of these anions) and very good recovery in real sample analysis. A similar nanoparticle was also reported by Ourari et al. [56] but with a remarkably lower LOD.

Tamiji and Nezamzadeh-Ejhieh [63] presented a carbon paste electrode (CPE) modified with Tin (II)-exchanged clinoptilolite NPs for BrO_3_^−^ detection. The best electrocatalytic activity of the electrode towards the analyte was obtained at a pH of 2 in an acidic medium. This sensor gave LOD and LDR of 0.06 and 5–100 µM, respectively. The electrode was further investigated for the effect of other oxidants on Br determination and the result showed that the presence of these oxidizing agents increased the maximum error involved in BrO_3_^−^ detection. A good recovery of BrO_3_^−^ in the spiked samples (well water, tap water, mineral water and bread) confirmed the practical usage of the electrode.

### 3.5. Bromate Determination by Modified Electrode with Quantum Dots

The biocompatibility and low cytotoxicity of carbon quantum dots (or carbon dots) (CQDs) have made them a worthy replacement for the metal-based quantum dots [64]. Carbon dots are fluorescent zero-dimensional materials with diameter < 10 nm. They have been applied in drug delivery, biosensors’ fabrication, bioimaging probes’ design and gene transmission. Their fluorescent properties have been tremendously exploited in analytical chemistry [65]. In addition, CQDs are easy to synthesize, can be made from cheap precursor, are chemically stable and water-soluble and therefore, stand a good chance as components of electrochemical sensors.

CQDs have been applied for the fabrication of sensors with low LOD and high sensitivity towards analytes, such as vitamins, polypeptides, DNA, hematin, drugs, water pollutants, acids and metal ions, among others [66]. These sensors are capable of analyte determination to a level as low as the femtomolar [67,68].

Xiang et al. [69] developed a very effective fluorescent probe for BrO_3_^−^ detection through the doping of silica nanoparticles with CQDs prepared from the pyrolysis of citric acid (CA). The florescence of the resultant probe was quenched by BrO_3_^−^ in an acidic medium. After the optimization of the electrolyte concentration, pH, temperature and reaction time, the sensor was applied for the analysis of BrO_3_^−^ and low LOD (1.1 ng mL^−1^) and relatively wide LDR (8–400 ng mL^−1^) were obtained. Real sample analyses of BrO_3_^−^ were carried out in drinking water samples with good recoveries.

Polyethyleneimine (PEI), an ionic polymer, was functionalized by Li et al. [70] with CQDs made from the pyrolysis of CA in order to use the photoluminescence (PL) sensor for BrO_3_^−^ detection. In order to obtain a very sensitive sensor, the experimental parameters were optimized. The interference study revealed that the electrode has a high selectivity for BrO_3_^−^ detection in the presence of interfering ions (ClO_3_^−^, SO_4_^2−^, Cu^2+^, Fe^3+^, CO_3_^2−^, Cl^−^, HPO_4_^2−^, AC^−^), with the exception of l^−^, IO_3_^−^, Cr_2_O_7_^2−^ and ClO^−^ that showed a noticeable interference effect. The resultant PL sensor gave a LOD of 0.17 nM over a linear range of 0.04–0.35 μM. Real sample application of the sensor was achieved with bottled, lake and drinking water, with good recovery. This sensor was also used for BrO_3_^−^ detection in pastry samples.

Liping and his group [71] developed a chemiluminescence (CL) sensor for BrO_3_^−^ with CQDs and sulfite. The CL peak obtained upon the injection of BrO_3_^−^ was used for quantifying the amount of BrO_3_^−^ in solution. The CL signal increased linearly within the range of 0.3–10 µM, with a LOD of 0.1 µM obtained. The mechanism of BrO_3_^−^ detection relied on the reaction between BrO_3_^−^, CQDs and sulfite in acidic medium, which led to the formation of hole- and electron-injected CQDs. The recombination of the duo brought about CL formation. This mechanism is in contrast with the assumption that energy transfer occurs between SO_2_^-^ and CQDs.

**Figure 6:**
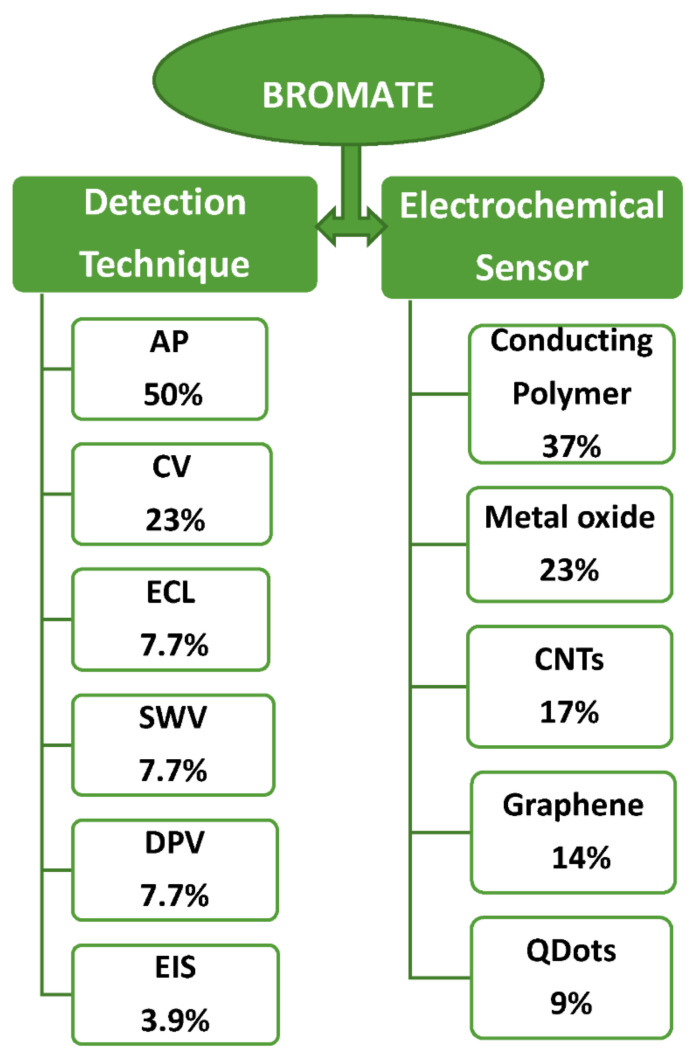
Summary of the electrochemical techniques and sensors for bromate detection.

Various electroanalytical techniques such as AP, CV, ECL, DPV and SWV have been deployed for BrO_3_^-^ detection as shown in Figure 6, with AP and CV been mostly used while EIS having the least. These techniques have been able to achieve the level of sensitivity and detection limit required for analysis of trace amounts of BrO_3_^−^, but the fact that EIS has not been utilized as much as these other techniques demands attention. Specifically, only one instance where EIS was used for BrO_3_^−^ was found in the literature (to the best of our knowledge) (Table 2).

The suitability of EIS for accurate determination of surface electrochemical processes through the measurement of the interaction of an analyte with the surface of a sensor made EIS one of the most popular electroanalytical techniques [11]. Besides, EIS is a very simple technique [12] that presents data in an easily comprehensible manner. It also helps in separating the charge transport associated with the bulk membrane from that of the interfacial reactions.

EIS has been successfully used for the determination of many analytes, such as iodate, chlorate, phthalates, perchlorate [7], amitrole, glyphosate [13], ascorbic acid (AA) [14], aldehyde [72], etc. A few of these are discussed here.

An EIS sensor for the detection of AA was developed by Qiu et al. [14] using Cu (I) catalyst. This sensor shows high selectivity, sensitivity and stability. LOD and LDR of 2.6 and 5–1000 pM respectively, were obtained. This sensor was applied on a real sample, such as urine, with a good recovery.

Glyphosate determination in water using a molecularly imprinted chitosan was performed by Fares et al. [13] with the aid of EIS. The sensor showed good selectivity and sensitivity towards glyphosate detection with a very low LOD (0.001 pg mL^−1^) over a wide LDR (0.31–50,000 pg mL^−1^). The selectivity of the technique was verified with the detection of various pesticides as interferents. Very high selectivity factors were obtained for glyfosinate (7.9), chlorpyrifos (43.5) and phosmet (14.5).

Boumya and his team [72] used the EIS technique for the determination of aldehyde at GCE. This EIS study revealed that the electrode is capable of detection over a LDR of 0.05–1000 µM with LOD of about 0.0109 µM. Real sample analyses of BrO_3_^−^ in orange juice, apple juice and drinking water were carried out with good recovery and standard deviation.

Importantly, the use of sensor material containing more than one nanomaterial has gone a long way in improving the electrochemical detection of bromate. Though a lot of materials have been used for the fabrication of sensors for BrO_3_^−^ detection, no such sensor was fabricated with the use of phthalocyanine or metal phthalocyanine, and only few with carbon quantum dots, despite their unique properties.

Meanwhile, there are many reports elucidating the applications of metal phthalocyanine with good sensitivity and low LOD, a few examples include the determination of carbohydrates [73], cresols, chlorophenols, phenols [74], toluene [75], hydrogen peroxide [76], hydrazine [77], glutathione [78], metronidazole [79], NADH [80], thiols [81], paracetamol [82], citrate [83], hydroxyquinoline [84] and hydrogen sulfide [85].

## 4. Conclusions

In this review, we have summarized the efforts made in electrochemical detection of bromate with high sensitivity and selectivity by modifying the electrode surface with different modifiers. We also pointed out those techniques and sensors that still need to be exploited for sensitive and selective determination of bromate, such as EIS and carbon quantum dots and metal phthalocyanine.

Owing to the greater advantages of EIS and the extraordinary properties of metal phthalocyanine and carbon quantum dots, more studies on the determination of bromate are expected in the near future based on the usage of EIS with regard to metal phthalocyanine and carbon quantum dots.

## Figures and Tables

**Table 1:** Standard potentials (E°) [16].

Reaction	E°/V (vs. NHE)
BrO_3_^−^ + 5H^+^ + 4e^−^ ↔ HBrO + 2H_2_O	1.450
BrO_3_^−^ + 6H^+^ + 5e^−^ ↔ ½Br_2_ + 3H_2_O	1.482
BrO_3_^−^ + 6H^+^ + 6e^−^ ↔ Br^−^ + 3H_2_O	1.423
HBrO + H^+^ + e^−^ ↔ ½Br_2_ + H_2_O	1.574
HBrO + H^+^ + 2e^−^ ↔ Br^−^ + H_2_O	1.331
Br_2_ + 2e^−^ ↔ 2Br^−^	1.087

**Table 2:** Different electrochemical methods and sensors for bromate detection.

Sensor				Linear Range	Detection Limit	
Category	Modified Electrode	Technique	Sample	µM	µM	Ref.
Conducting	PEDOT/SiMo_12_/GCE	AP		30–8 × 10^3^		[3]
Polymer	PTh-D/nafion/AuE	ECL	Water	1–1 × 10^5^	1	[19]
	Fe(III)P/MWCNT/GCE	AP		2–150	0.6	[20]
	Fe(III)P/MWCNT/SPCE	AP	Water	0.1–2.5	0.043	[21]
	Ni/POM/Ppy/GCE	EIS	Water	100–2 × 10^3^	0.2	[23]
	Nd(SiMo_7_W_4_)_2_/PPy/GCE	CV		0.001–0.032		[24]
	Nafion/Ti_3_C_2_T_x_/GCE	DPV	Water	0.05–5	0.041	[30]
	MWCNT_5_/PDDA/PMo_12_/PGE	AP	Water	0.05–0.4	0.020	[31]
	TMOPP-Mn(III)Cl)/GE	SWV	Bread	0.1–1 × 10^4^	0.004	[32]
Carbon	PMo_12_/MWCNTs/PGE	AP		5–15 × 10^3^	0.50	[36]
nanotubes	SWCNT/Os(III)/GCE	AP		1–2 × 10^3^	0.036	[37]
	*f*-MWCNT–P-L-His–ZnO/GCE	AP	Water	2–15 × 10^3^	0.30	[38]
	SiMo_12_O_40_^4−^/[Cu(bpy)_2_]^2+^/CNT/GCE	AP		0.01–20	0.001	[39]
	MWCNT/Pd/GCE	AP		100–40 × 10^3^		[40]
	MWCNT/PLL/Hb/GCE	AP	Water	15–6 × 10^3^	0.96	[41]
Graphene	GO-PdNPs/GCE	AP	Bread	1–1 ×10^3^	0.105	[11]
	Graphene-β-CD/GCE	AP	Water	0.1–177	0.033	[48]
	rGO-PDDA/PMo_12_/GCE	CV		20–10 × 10^3^		[16]
Metal (oxide)	CuII-DHB/CPE	AP		2–14 × 10^3^	0.010	[56]
	Cd-IL/CPE	CV		0.005–0.020	0.003	[57]
	Ag/PMo_12_/PBz/GCE	AP			0.086	[58]
	Pd-NPs/PANI/SBA-15/GCE	CV		8–40 × 10^3^	5	[60]
	AuRh/GCE	CV		1–26	1	[61]
	CuO/FIA/SPCE	CV		0.066–1990	0.027	[62]
	CNP-Sn(II)/CPE	SWV	Water	5–100	0.060	[63]
Carbon dots	CDs-PEI	ECL	Water	0.04–0.35	0.0002	[70]

## Data Availability

Not applicable but for other contents request can be made directly to the authors.

## Abbreviations

AA	Ascorbic acid
AP	Amperometry
BrO_3_^−^	Bromate
CNTs	Carbon nanotubes
CQDs	Carbon quantum dots
CV	Cyclic voltammetry
DA	Dopamine
ECL	Electrochemiluminescence
EIS	Electrochemical impedance spectroscopy
GC	Gas chromatography
GCE	Glassy carbon electrode
HPLC	High-performance liquid chromatography
IC	Ion chromatography
LC	Liquid chromatography
LDR	Linear dynamic range
LOD	Limit of detection
MP	Metal phthalocyanine
MWCNT	Multi-walled carbon nanotube
PFA	Polyunsaturated fatty acid
Rgo	Reduced graphene oxide
SP	Spectrophotometry
SPCE	Screen-printed carbon electrode
SWV	Square wave voltammetry
